# Lactylation may be a Novel Posttranslational Modification in Inflammation in Neonatal Hypoxic-Ischemic Encephalopathy

**DOI:** 10.3389/fphar.2022.926802

**Published:** 2022-06-02

**Authors:** Yue Zhou, Li Yang, Xiaoying Liu, Hao Wang

**Affiliations:** Department of Pharmacy, Xindu District People’s Hospital of Chengdu, Chengdu, China

**Keywords:** lactylation, inflammation, hypoxic-ischemic encephalopathy, macroglia, lactyl-CoA

## Abstract

Perinatal hypoxia-ischemia remains the most common cause of acute neonatal brain injury and is associated with a high death rate and long-term neurological abnormalities such as memory and cognitive deficits and dyskinesia. Hypoxia-ischemia triggers an inflammatory cascade in the brain that is amplified by the activation of immune cells and the influx of peripheral immune cells into the brain parenchyma in response to cellular injury. Thus, acute cerebral hypoxic-ischemic inflammation is a major contributor to the pathogenesis of newborn hypoxic-ischemic brain injury. Lactate is a glycolysis end product that can regulate inflammation through histone lactylation, a unique posttranslational modification that was identified in recent studies. The purpose of this review is to outline the recent improvements in our understanding of microglia-mediated hypoxic-ischemic inflammation and to further discuss how histone lactylation regulates inflammation by affecting macrophage activation. These findings may suggest that epigenetic reprogramming-associated lactate input is linked to disease outcomes such as acute neonatal brain injury pathogenesis and the therapeutic effects of drugs and other strategies in relieving neonatal hypoxic-ischemic brain injury. Therefore, improving our knowledge of the reciprocal relationships between histone lactylation and inflammation could lead to the development of new immunomodulatory therapies for brain damage in newborns.

## Introduction

Approximately four million newborns worldwide have died as a result of perinatal hypoxia. Hypoxic-ischemic encephalopathy (HIE) affects between 0.5 and 2/1000 full-term neonates in developed countries and is one of the most frequently documented causes of neonatal death and poor neurodevelopmental outcomes (Low and Research, 2004; Blair and Watson, 2006). Perinatal hypoxic-ischemic (HI) brain damage has become a global public health issue with significant societal costs. After acute hypoxia-ischemia, brain damage occurs in four separate phases (acute insult, the latent period, the secondary phase, and the tertiary phase), which develop over several weeks (Low and research, 2004). Oxidative stress, cerebral reperfusion/reoxygenation, glutamatergic excitotoxicity, inflammation and severe mitochondrial dysfunction are key pathophysiological determinants of HIE-related brain injury (Perlman, 2006; Hassell et al., 2015). Epilepsy, cerebral palsy, motor and cognitive decline, attention-deficit hyperactivity disorder, and behavior difficulties are lifelong neurological impairments associated with HIE (Sävman et al., 2010; Millar et al., 2017). Currently, Apgar scores, analysis of umbilical cord and peripheral blood acid levels, magnetic resonance imaging, and amplitude integrated electroencephalography are used to diagnose and determine the clinical prognosis of brain injury (Pfister and Soll, 2010; Millar et al., 2017).

Inflammation has long been thought to play a role in the pathogenesis of cerebral HI injury (Li et al., 2017). Hypoxia-ischemia causes rapid and powerful activation of resident brain cells, as well as circulating peripheral leukocyte infiltration. The initial inflammatory response to hypoxia-ischemia causes subsequent neuronal injury that can persist for many days before being resolved by the anti-inflammatory response (Basha et al., 2014; Hagberg et al., 2015). A number of experimental studies have demonstrated the critical roles of resident immune cells in enhancing brain damage and subsequent tissue repair and remodeling during the different stages of HI pathogenesis (Hagberg et al., 2012; Basha et al., 2014). However, the specific inflammatory processes that occur in the brains of young patients after hypoxia-ischemia are still largely unclear.

Previous studies have reported that extracellular lactate can stimulate histone lactylation and that histone Kla (lysine lactylation) levels are increased by glucose (from which most intracellular lactate is derived) in a dose-dependent fashion (Zhang et al., 2019). In accordance with this finding, inhibitors of intracellular lactate production were found to reduce histone Kla levels, whereas a compound that inhibits glycolysis (thus increasing intracellular lactate concentrations) was observed to increase histone Kla levels. Hypoxia, which increases the levels of intracellular lactate by enhancing glycolysis, is associated with an increase in histone Kla levels (Zhang et al., 2019).

Lactate is a glycolytic product and a substantial energy source produced after hypoxia. However, glycolysis inhibitors can diminish lysine lactylation by depleting lactate, and mitochondrial inhibitors or hypoxia can amplify lysine lactylation by enhancing lactate production, indicating that both exogenous and endogenous lactate contribute to lysine lactylation (Zhang et al., 2019). In addition, lactate affects gene transcription through histone lactylation, which is a unique posttranslational modification (Liberti and Locasale, 2020). HIE is frequently linked to metabolic activity imbalance and the production of excessive lactate. Lactate suppresses signaling pathways and modifies histones to decrease inflammatory macrophage activation and promote homeostatic M2-like macrophage polarization through various mechanisms in certain subcellular regions (Zhang et al., 2019; Ivashkiv, 2020). We aim to present an overview of metabolic changes and lactate production after HIE, as well as a description of a newly discovered lactate-induced histone modification in this review. Furthermore, we explore the role of histone lactylation in neonatal brain injury by focusing on inflammation and macrophage activation and evaluate the therapeutic potential of targeting histone lactylation in neonatal brain injury.

## Changes in Metabolic Status in Hypoxic-Ischemic Encephalopathy

Metabolism is required for most cellular processes, including ATP synthesis; cell division; synaptogenesis; neurotransmitter release and uptake; ionic gradient maintenance; the synthesis of proteins, nucleic acids, and carbohydrates; and mitochondrial function (Dienel, 2019). The developing brain uses glucose and its substrates, such as ketones, fatty acids, and glycerol, to meet its high metabolic demands, and the adult brain uses glucose as its principal energy substrate (Takahashi et al., 1999). Although the amount of glucose oxidized *via* the TCA cycle as an energy supply is lower in the developing brain, glucose metabolism *via* the pentose phosphate pathway is enhanced in adults and provides materials necessary for nucleotide synthesis, as well as lipid biosynthesis and glutathione reduction (Morken et al., 2014; Brekke et al., 2015; Brekke et al., 2017).

HIE is caused by a reduction in oxygen and nutrient delivery to the brain, lactic acidosis and CO_2_ clearance (Fatemi et al., 2009). Deep gray matter injury, including damage to the thalamus, hippocampus, putamen, and basal ganglia, as well as cortical loss can be revealed by magnetic resonance imaging (MRI) and proton magnetic resonance spectroscopy (1H-MRS) (Fatemi et al., 2009; Higgins et al., 2011). After neonatal and pediatric brain injury, acute changes in energy metabolism and long-term metabolic dysregulation leave the brain susceptible to damage and unable to maintain the numerous processes required for normal development (Goergen et al., 2014; Kendall et al., 2014; Janjic et al., 2020). Any injury compounded by the rapidly developing brain’s high metabolic demands can jeopardize normal developmental processes and result in neurodevelopmental consequences ranging from mild to severe learning impairments (Shanmugalingam et al., 2006; Goergen et al., 2014). Furthermore, a number of investigations have shown that metabolite ratios provide diagnostically useful information and can predict neurological prognosis (Shanmugalingam et al., 2006; Goergen et al., 2014). The strongest predictor of outcome was recently revealed to be a combination of lactate/N-acetylaspartate and arterial spin labeling (ASL) perfusion MRI data (De Vis et al., 2015). The presence of lactate/choline in basal nuclei (Barkovich et al., 1999) and lactate/N-acetylaspartate in basal nuclei accurately predicted worse neurodevelopmental outcome at 1 year with high specificity and predictive value (Amess et al., 1999), and excessive lactate in the brain is linked to poor outcomes (Cady, 2001; Oz et al., 2014).

## Modifications to Histone

The core nucleosome is the most basic unit of chromatin and is made up of four pairs of histones (H2A, H2B, H3, and H4) wrapped around the DNA double helix (Xu et al., 2021). Positively charged amine groups on lysine residues in histones can be found within catalytic sites or, more commonly, on the solvent-exposed protein surface. Thus, the lone-pair electrons of the amine of lysine along with the preferential location of the amine group on the protein surface render lysine vulnerable to a variety of modifications (Luo, 2018). Histones comprise chromatin-bound proteins (called the nucleosome core) and DNA that regulate gene expression through various posttranslational pathways. Classic posttranslational modifications, such as acetylation, malonylation, and succinylation, disturb the spatial structure of proteins to control a variety of physiological and biochemical activities in cells (Tarazona and Pourquie, 2020; Gupta et al., 2021). Propionylation (Chen et al., 2007), butyrylation (Zhang et al., 2009), crotonylation (Wang et al., 2021), malonylation (Xie et al., 2012), succinylation (Mu et al., 2021), glutarylation (Bao et al., 2019), 2-hydroxyisobutyrylation (Dai et al., 2014), hydroxybutyrylation (Xie et al., 2016), and lactonylation (Zhang et al., 2019) are nine novel types of histone lysine (K) acylation that were detected by high-resolution mass spectrometry-based proteomics analysis. These novel histone modifications differ from histone lysine acylation in terms of hydrocarbon chain length, hydrophobicity, and charge and drastically increase the diversity of histone modifications. Most of the novel acylation sites overlap with histone lysine acylation sites, indicating a synergistic effect. However, some sites have nonoverlapping alteration patterns, which could allow diverse epigenetic outcomes.

## Discovery of Histone Lactylation

Lactylation was proposed as a new histone acylation modification in 2019 (Zhang et al., 2019). Mass spectrometry was used to identify the four histones associated with lysine lactylation in HeLa cells and bone marrow-derived macrophages (BMDMs), and twenty-eight lysine lactylation sites on core histones were discovered (Zhang et al., 2019). In addition, lactate inhibition was found to suppress the utilization of glucose-derived carbon for lysine lactylation not lysine acetylation using the tracer 13C-glucose. Lysine lactylation is reduced when lactate dehydrogenase (LDH) is genetically depleted (Zhang et al., 2019). Researchers have discovered that when human cell lines are exposed to substances or conditions that elevate cellular lactate levels, histone lactylation is increased (Pan et al., 2022). Based on these findings, a novel histone modification specifically modulated by lactate was identified, suggesting that lysine lactylation is controlled by changes in the metabolic dynamics of glucose and lactate levels. Conversely, lactate regulates histone lactylation, which is a byproduct of glycolysis.

## Involvement of Lactyl Coenzyme A in Lysine Lactylation

Both the lysine acetyltransferase (KAT) enzyme P300 and lactyl-CoA are required for enzymatic lysine lactylation (Zhang et al., 2019). Despite this, there is no evidence of a lactyl-CoA synthetase or transferase that converts lactate to lactyl-CoA in mammals. According to recent research, lactyl-CoA is much less abundant than other acetyl-CoAs, such as acetyl-CoA, succinyl-CoA, and propionyl-CoA (Varner et al., 2020). As a result, a reduction in lactyl-CoA levels could limit the rate of enzymatic lysine lactylation. Lactylation is an attractive link between metabolism and cell signaling because it is modulated under critical physiological conditions, including cancer and immune cell activation (Trefely et al., 2020). However, more research into the biochemistry and physiological roles of lactylation is needed. In addition, lactyl-CoA and acetyl-CoA levels may be indicative of histone lactylation and acetylation levels, respectively, the ratio of which influences pyruvate outflow and malignant cell fate.

## Nonenzymatic Lysine Lactylation

Lysine acylation has been demonstrated to result from a nonenzymatic reaction with lactoylglutathione (LGSH), in addition to the enzyme-dependent transfer of a lactyl group from lactyl-CoA to lysine residues (Gaffney et al., 2020). The glycolytic byproduct methylglyoxal attaches to glutathione *via* the thioesterase glyoxalase I (GLO1) to produce LGSH, which is hydrolyzed to glutathione and D-lactate by GLO2. Recently, studies have shown that transferring a nonenzymatic lactyl group from LGSH to protein lysine residues results in a “LactoylLys” alteration in proteins, which can be blocked by GLO2 and affects glycolytic activity (Gaffney et al., 2020). Furthermore, nonenzymatic lysine acetylation of mitochondrial proteins by acetyl-CoA and acetylglutathione is increased through a proximal S-acetylated thiol intermediate, which is blocked by GLO2 (James et al., 2017).

## The Contribution of Lactylation to Hypoxic-Ischemic Encephalopathy-Associated Inflammation

### Microglia are Innate Immune Cells in the Brain

Microglia are derived from a type of erythromyeloid progenitor (Matcovitch-Natan et al., 2016), and constitute a large portion of the glial population in the developing periventricular white matter, accounting for up to 20% of the total glial population. Microglia rarely need to be replaced by the BM under normal conditions (Perry et al., 2010). Although both microglia and macrophages express CD11b, microglia express intermediate levels of CD45 (CD11b+/CD45int), while macrophages express high levels of CD45 (CD11b+/CD45hi). Furthermore, these cells express low levels of MHC-II, indicating that they are not fully differentiated (Li et al., 2017).

Neonatal microglia are slightly more active under normal conditions than adult microglia and express higher levels of MHC-II and costimulatory markers, including CD86 and CD40. Postnatal microglia also respond differently to macrophage colony-stimulating factor and granulocyte-macrophage colony-stimulating factor in terms of proliferation and are less reliant on astrocytes for expansion than other cells (Santambrogio et al., 2001). Microglia are critical myeloid cells in the central nervous system (CNS) that serve as the first and most significant line of defense against invading pathogens and environmental toxins. Resting microglia have highly ramified and motile processes in the healthy state, generating a 3D nonoverlapping network that is necessary for CNS immunosurveillance of particular domains (Ransohoff and Perry, 2009). Microglia play an active role in synaptic pruning and neurogenesis in the developing brain, as well as maintaining homeostasis (Matcovitch-Natan et al., 2016).

### Inflammation

Microglia are the first cells in the developing brain to respond to HI damage. They have a ramified shape and extend and retract their cellular processes to monitor the brain microenvironment continuously under healthy conditions. When microglia are activated, they transform into amoeboid-like cells with immunological functions, including the ability to produce cytokines and matrix metalloproteinases, phagocytic ability, and the ability to present antigens (Algra et al., 2013; Boche et al., 2013). Activated microglia travel to the injury site and release proinflammatory substances that damage the BBB, allowing peripheral leukocytes to enter the cerebral parenchyma (Leonardo and Pennypacker, 2009; Iadecola and Anrather, 2011). In addition, glial cells, invading monocytes, and T cells contribute to the inflammatory response by producing cytokines, chemokines, reactive oxygen species (ROS), and excitatory neurotransmitters (Liu and Mccullough, 2013; Hagberg et al., 2015; Ziemka-Nalecz et al., 2017).

**FIGURE 1:**
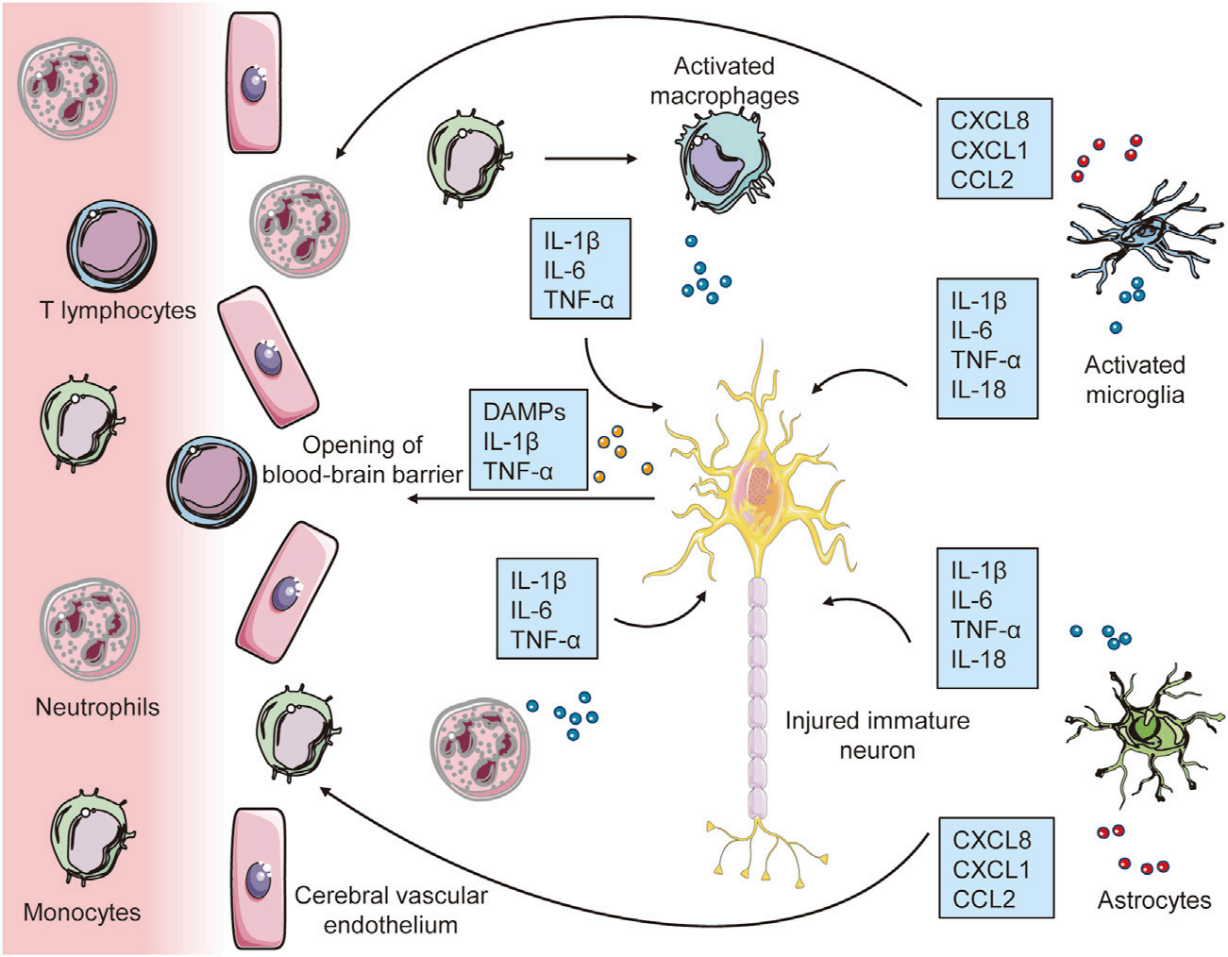
The early inflammatory response to hypoxia-ischemia in the developing brain. Immune cells (microglia, macrophages and astrocytes) first sense these damage signals through TLRs and cytokine receptors, which lead to inflammatory activation of microglia and astrocytes. By releasing a significant number of proinflammatory cytokines, activated glial cells have a direct neurotoxic effect in initiating damage to immature neurons. Furthermore, the synthesis and release of chemokines by brain resident immune cells and other processes etc, in conjunction with molecular pattern molecules (DAMPs) released by injured neurons, increase blood–brain barrier (BBB) permeability, which leads to the recruitment of peripheral immune cells to the injured brain, contributing to further exacerbation of neuroinflammation and subsequent neuronal injury.

Macrophages suppress inflammation by modifying tissue and eliminating cellular detritus. These cells are classified into two subtypes: proinflammatory (M1) macrophages and anti-inflammatory (M2) macrophages. During energy failure, microglia tend to be polarized toward the M1 phenotype after hypoxia-ischemia, and M1 microglia release proinflammatory cytokines, complement factors, proteases, and excitotoxic amino acids, which have toxic effects on neurons and glia in the surrounding area (Rocha-Ferreira and Hristova, 2015). Furthermore, excessive release of ROS and NO by activated microglia can cause oxidative damage in the developing brain (Kaur et al., 2013). The release of IL-1β and IL-18 is one of the most powerful inducers of microglial M1 polarization. Previous studies have shown that in animal models, the anti-inflammatory medication minocycline can inhibit microglial responses and significantly ameliorate neonatal HI brain injury (Arvin et al., 2002). These findings suggest that initial inflammatory responses in microglia enhance neonatal HI brain injury. As a result, it is believed that reducing microglial M1 polarization may be beneficial for the treatment of newborn HI brain injury Figure 1.

Microglia have also been implicated in the alleviation of inflammation and reparative processes following acute HI brain damage. During the delayed phase after newborn stroke, phagocytosis of debris by activated microglia is crucial for repairing the injured brain (Faustino et al., 2011). In neonatal and adult stroke models, selective ablation of microglia increases the levels of proinflammatory cytokines and chemokines and exacerbates brain injury (Lalancette-Hebert et al., 2007; Faustino et al., 2011). In an adult stroke model, injection of microglia was found to protect neurons from ischemia-induced neuronal damage by maintaining brain-derived neurotrophic factor levels (Hayashi et al., 2006). In addition, a number of stroke studies in adults suggest that after the initial M1 polarization of microglia following ischemia, microglia switch to an anti-inflammatory M2 phenotype, resulting in inflammation resolution, debris clearance, and tissue restoration (Hu et al., 2012; Patel et al., 2013). However, the mechanisms underlying the resolution of the proinflammatory phase in the injured brain following neonatal hypoxia–ischemia are still unknown.

### Involvement of Histone Lactylation in Inflammation

**FIGURE 2:**
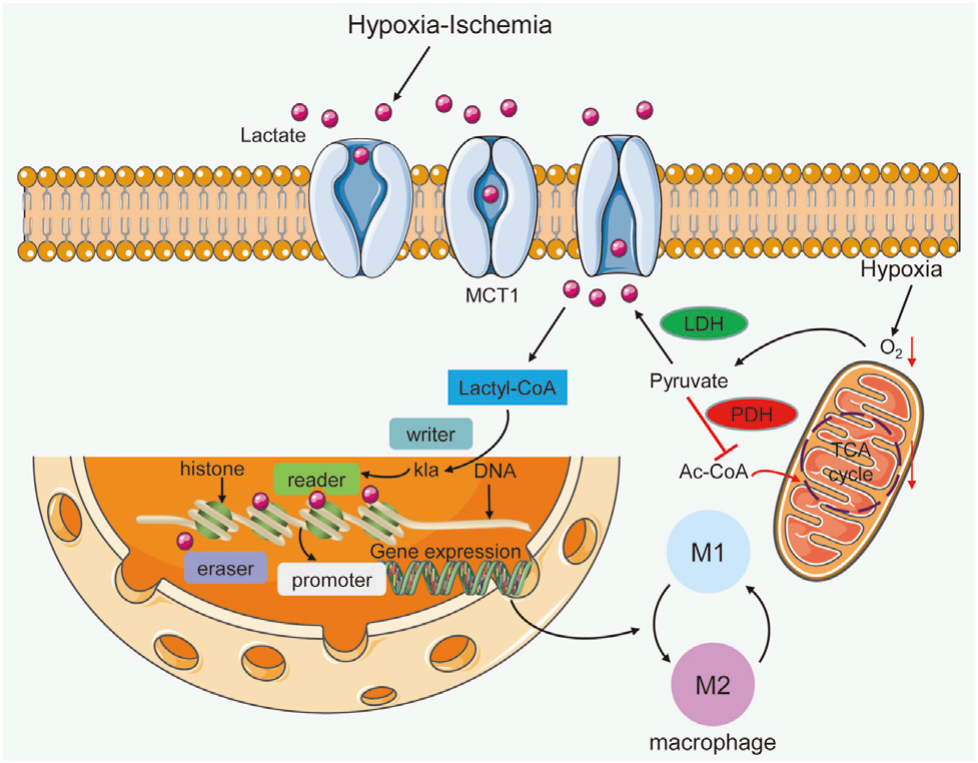
Lactate acts as a signaling molecule in macrophages to trigger epigenetic changes caused by histone lysine lactylation. Hypoxia-ischemia can increase lactate production and entry into macrophages. In addition, hypoxia triggers the promotion of lactate by increasing glycolysis and inhibiting the Krebs cycle. Lactyl-CoA synthetase converts lactate to lactyl-CoA and further induces histone kla at the promoter, which is controlled by writer, eraser and reader enzymes. In gene promoter regions, histone kla can regulate gene transcription and cause alterations in M1 and M2 macrophages.

Lactate inhibits signaling pathways and alters histones to decrease inflammatory macrophage activation and promote homeostatic M2-like polarization (Zhang et al., 2019; Ivashkiv, 2020). Toll-like receptors (TLRs) on macrophages identify bacterial pathogens, causing early inflammation and pathogen clearance. Reports have shown that the TLR signaling adaptor B-cell adapter for PI3K (BCAP) inactivates glycogen synthase kinase 3 (GSK3) and forkhead box protein O1 (FOXO1) and modulates the transition of macrophages from an inflammatory to a reparative phenotype by boosting histone lactylation (Irizarry-Caro et al., 2020). Previous studies have shown that histone lactylation induces M2-like gene expression in the late phase of M1 macrophage polarization after the inflammatory response to repair damage *via* the putative histone lactylation writer protein p300 (Zhang et al., 2019). In addition, lactate could induce histone lactylation through p300 and enhance the expression of numerous profibrotic genes im macrophages in the lungs (Cui et al., 2021). RNA sequencing was used to determine the functional purpose of lactylation, and it was found that the expression of genes enriched in wound healing, particularly homeostasis genes that are important for maintaining homeostasis and are linked to M2-like macrophages, was significantly enhanced by histone lactylation. The administration of exogenous lactate to M1 macrophages results in enrichment of lysine lactylation at the Arg1 (arginase 1, a M2 marker gene) promoter and enhances gene expression. Similarly, loss of LDHA in M1 macrophages reduces histone lactylation and Arg1 expression (Irizarry-Caro et al., 2020). Since Arg1 is expressed under proinflammatory conditions, this gene, which is the key modulator of arginine metabolism in M2 macrophages, is generally used as a hallmark gene of the alternative immune response (Zhang et al., 2019). These findings demonstrate that lactate is required for proper histone lactylation, which stimulates gene expression and promotes M2 polarization following M1 macrophage polarization, eventually restoring homeostasis. Finally, lactate accumulation throughout metabolic processes is used as a predictor of histone lactylation, which governs M1 macrophage homeostasis (Zhang et al., 2019). However, these discoveries have sparked many doubts, and further research is needed. To identify new therapeutic targets and strategies for resolving inflammation, further research on histone lactylation is needed Figure 2.

### Lactate and Lactylation Dynamics

Macrophage phenotypic transformation is a dynamic process resulting from a changing environment (Toledo and Pioli, 2019). H3K18la (histone three lysine 18 sites lactylation) levels are relatively high at the promoters of genes that are activated at later time points and encode noninflammatory, homeostatic mediators (Arg1), while inflammatory response genes (such as nitric oxide synthase 2) is induced early (4 h) in mouse BMDMs treated with LPS and IFN-γ or with gram-negative bacteria, as expected (Zhang et al., 2019). Histone lactylation induction (24 h) takes longer than histone acetylation induction (6 h) in lung fibrosis, and histone lactylation levels are elevated in the promoter regions of genes involved in biological activities, such as wound healing, suggesting that the lactylation of histones is physiologically relevant. During macrophage activation, exogenous lactate boosts the expression of several anti-inflammatory and proangiogenic genes, including VEGFA, in addition to enhancing the expression of Arg1 (Irizarry-Caro et al., 2020). Thus, M1 polarization increases lactate levels and the activation of the endogenous “lactate clock,” which is usually delayed, as well as the expression of M2-like genes involved in inflammatory repair in macrophages, which influences downstream mechanisms that regulate homeostatic responses (Cui et al., 2021).

Lactate and lactylation may have different roles under different pathophysiological conditions. More research is needed to determine the specific temporal relationship between the lactate level and histone lactylation and other interconnected metabolite-epigenetic interactions. The mechanisms controlling lactate concentrations in microdomains within the nucleus, the histone lactylation concentration needed to drive epigenetic changes, and the mechanisms by which fluctuations in lactate levels control the expression of specific genes are all unknown (Chen et al., 2021). It will be fascinating to see if lactate homeostasis can be restored once these questions are answered.

## Histone Lactylation-Targeting Drugs

Several medications that target histone posttranslational modifications have been clinically tested and have shown significant therapeutic effects in a variety of disorders. For example, drugs that reduce histone methylation by suppressing histone methyltransferases, those that reduce histone deacetylation by suppressing HDACs, and those that increase histone crotonylation are all beneficial in kidney injury (Fontecha-Barriuso et al., 2018). Similar to p300, class I HDAC inhibitors and activators affect various physiological processes that are regulated by histone lactylation and can be used to treat various disorders. For example, lactate improves ferroptosis resistance in tumor cells by stimulating the AMPK-SREBP1-SCD1 pathway, but whether histone lactylation plays a role in this process is unknown (Zhao et al., 2020). Strategies for controlling the glycolytic swing from lactate production, histone lactylation to acetyl-CoA production and the TCA cycle represent novel targeted therapies for HIE (Dai et al., 2022). Thus, further research on the entire mechanisms of histone lactylation is needed to uncover more targets that are useful for therapeutic research and development.

## Conclusion

According to a large body of evidence, brain inflammation is a crucial contributor to HIE pathogenesis and is involved in both early and late brain injury following HI, as well as the repair and recovery of brain tissues. Following energy deprivation, several immune cells and chemical mediators of inflammation, such as cytokines, chemokines, and adhesion molecules, are implicated in the brain-immune interaction. If acute inflammation is not appropriately addressed, it can progress to chronic inflammation, which can harm the developing brain and make it more vulnerable to HI injury.

Previous studies on lactate are at the vanguard of efforts to delineate the processes by which nutritional cues are integrated with metabolite variations and promote epigenetic programming (Chisolm and Weinmann, 2018). The mechanisms underlying inflammation have been clarified in research on lactylation in macrophages. It will be fascinating to learn more about how lactate metabolism affects epigenetic programming during inflammation in future studies. More research is needed to apply the findings to infants with newborn HI brain injury. The study of histone lactylation regulation is expected to lead to a promising therapeutic strategy for HIE.
